# Cell death inhibition by KSHV

**DOI:** 10.18632/aging.100829

**Published:** 2015-10-22

**Authors:** Vinay Murtadak, Christoph Becker, Michael Stürzl

**Affiliations:** Division of Molecular and Experimental Surgery, Translational Research Center Erlangen, Department of Surgery, 91054 Erlangen, Germany

**Keywords:** KSHV, HHV-8, cell death, apoptosis, necroptosis

Kaposi's sarcoma-associated herpesvirus (KSHV) is the causative agent of the endothelial cell-derived tumor Kaposi's sarcoma and of the lymphoproliferative disorders primary effusion lymphoma and multicentric Castleman's disease. The life cycle of KSHV includes a latent and a lytic/productive phase that both contribute to tumorigenic activity. Major threats for infected tumor cells are attacks of the immune system and the induction of endogeneous cell death programs in response to the virus infection. KSHV opposes these threats for example by hiding from the immune attack by entering the latent infection phase where only few genes are expressed and replication is absent or in the productive phase by the downregulation of major histocompatibility complex class I on the surface of infected cells. Moreover, KSHV uses several different strategies to counteract endogenous cell death programs such as apoptosis and necroptosis.

KSHV encodes more than 87 ORFs (open reading frames) and numerous ribosomal-loaded not previously annotated RNAs [1]. ORF activities on key survival mechanisms such as activation of NF-κB [2], inhibition of p53 [3] and inhibition of foreign DNA sensing [4] were analysed in systematic transfection analyses applying a validated KSHV expression library [5]. A high throughput transfection approach in the microscale format [reversely transfected cell microarrays (RTCM)] showed that the latently expressed K13/vFLIP gene is the major NF-κB activator encoded by KSHV. Moreover, the viral tegument protein ORF75 was identified as a new NF-κB activating protein which is able to cooperate with K13/vFLIP. Both genes activated the classical NF-κB pathway but used different routes downstream of TAK1 [2]. K13/vFLIP activates NF-κB by interacting with cytosolic IKK-γ. This results in the inhibition of apoptosis by subsequent activation of the expression of inhibitor of apoptosis proteins (IAPs). Moreover, an analysis of the K13/vFLIP-associated proteome in endothelial cells with two-dimensional fluorescence difference gel electrophoresis showed that manganese superoxide dismutase (MnSOD) is robustly induced by the NF-κB-activating function of K13/vFLIP [6]. MnSOD efficiently inhibited apoptosis induced by reactive oxygen species, which may be an important survival mechanism in the inflammatory environment of KS. Other studies suggested that K13/vFLIP may inhibit apoptosis by blocking caspase-8 activation in the apoptotic death-inducing signaling complex (DISC). In contrast it was hypothesized that K13/vFLIP may induce necroptosis in infected cells [7] because insufficient caspase 8 activity has been identified as a key parameter in the induction of necroptosis [8]. However, data from ongoing in vitro studies in our laboratory indicate that a latent KSHV infection where K13/vFLIP is one of the few expressed viral genes induces resistance of infected cells to experimentally induced necroptosis (unpublished observation), indicating that K13/vFLIP may rather act on the pro-survival side.

In another RTCM-based study the effects of all KSHV-encoded genes on the inhibition of p53 were investigated. The tumor suppressor p53 is a major regulatory molecule of apoptosis and cell cycle progression. Three structural proteins namely ORF22 (envelope glycoprotein gH), ORF25 (major capsid protein) and ORF64 (tegument protein) were identified as potent inhibitors of p53 signaling [3]. All three of these genes inhibited p53-mediated apoptosis in response to Nutlin-3 treatment in non-infected and KSHV-infected cells. Interestingly, all three genes could inhibit the p53-mediated induction of BAX and PIG3, which are known to be key mediators of mitochondrial apoptosis and the DNA damage response pathway, respectively [3].

Finally, viral DNA establishes a pathogen associated molecular pattern (PAMP) that can trigger the host innate immune responses by stimulating production of type I interferons (IFNs). This leads to repression of viral replication and elimination of virus infected cells by apoptosis. Systematic analyses of all KSHV-encoded genes identified the tegument protein ORF52 as a potent inhibitor of this pathway [4]. ORF52 acted by binding to viral DNA and to the host cytosolic DNA sensor cyclic GMP-AMP synthase (cGAS), directly inhibited cGAS enzymatic activity and antagonized the cGAS-dependent DNA sensing in infected cells [4].

Altogether, the transfection studies summarized here indicate that KSHV encodes a large repertoire of genes regulating survival of the infected cell in all stages of the viral life cycle (Figure 1). In the early infection (entry phase) tegument proteins like ORF52, ORF75 and ORF64 may be released from the virus and counteract cellular suicide programs. Other cell death opposing proteins present in the virus particle may likely not enter the cell (envelope protein ORF 22) or may be trapped (capsid protein ORF25) in the virus structure. In the latent phase K13/vFLIP and in some cell types also ORF75 (expressed in latently infected HEK293 cells [2]) may ensure cell survival. In the lytic/productive phase ORF22, ORF25, ORF52, ORF64 and ORF75 may regulate cell survival to obtain an optimal virus yield. These transfection-based results require validation in the whole virus context applying recombinant virus approaches. However, it is apparent that KSHV carries a broad anti-cell death repertoire, which may provide relevant targets for anti-viral therapy strategies.

**Figure 1:**
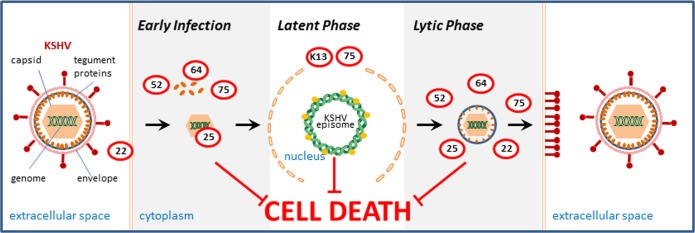
KSHV encodes a large repertoire of genes regulating survival of the infected cell in all stages of the viral life cycle. Genes identified by systematic transfection analyses to counteract cell death are given in red circles (ORF numbers are given). ORFs 22 and 25 may not enter the cell or may be trapped in the viral capsid in the early infection but are expressed in the infected cell and may be active in the lytic phase.

## References

[1] Arias C (2014). PLOS Pathogens.

[2] Konrad A (2009). J. Virol.

[3] Chudasama P (2015). Oncogene.

[4] Wu JJ (2015). Cell Host Microbe.

[5] Sander G (2008). J. Virol.

[6] Thurau M (2009). J. Virol.

[7] Feoktistova M (2012). Cell Cycle.

[8] Günther C (2011). Nature.

